# Analyses of the photosynthetic characteristics, chloroplast ultrastructure, and transcriptome of apple (*Malus domestica*) grown under red and blue lights

**DOI:** 10.1186/s12870-021-03262-5

**Published:** 2021-10-23

**Authors:** Zhiqiang Li, Qiaojing Chen, Youyan Xin, Zhuoxin Mei, Aiyun Gao, Wenjun Liu, Lei Yu, Xuesen Chen, Zijing Chen, Nan Wang

**Affiliations:** 1 State Key Laboratory of Crop Biology, College of Horticulture Science and Engineering, Shandong Agricultural University, 271018 Tai’an, Shandong China; 2 Collaborative Innovation Center of Fruit & Vegetable Quality and Efficient Production, 271000 Tai’an, Shandong China

**Keywords:** Apple, Red and blue light, Growth and development, Carbon and nitrogen metabolism

## Abstract

**Background:**

Light quality significantly affects plant growth and development, photosynthesis, and carbon and nitrogen metabolism. Apple (*Malus domestica* Borkh.) is a widely cultivated and economically important fruit crop worldwide. However, there are still few studies on the effects of different light qualities on the growth and development of apple seedlings.

**Results:**

In this study, we explored the effects of blue and red light treatments on the growth and development, photosynthetic characteristics, leaf chloroplast ultrastructure, and carbon and nitrogen metabolism of apple seedlings. Blue light significantly inhibited apple plant growth and leaf extension, but it promoted the development of leaf tissue structures and chloroplasts and positively affected leaf stomatal conductance, the transpiration rate, and photosynthetic efficiency. The red light treatment promoted apple plant growth and root development, but it resulted in loosely organized leaf palisade tissues and low chlorophyll contents. The blue and red light treatments enhanced the accumulation of ammonium nitrogen in apple seedlings. Moreover, the blue light treatment significantly promoted nitrogen metabolism. Additionally, an RNA-seq analysis revealed that both blue light and red light can significantly up-regulate the expression of genes related to carbon and nitrogen metabolism. Blue light can also promote amino acid synthesis and flavonoid metabolism, whereas red light can induce plant hormone signal transduction. The expression of a gene encoding a bHLH transcription factor (MYC2-like) was significantly up-regulated in response to blue light, implying it may be important for blue light-mediated plant development.

**Conclusions:**

Considered together, blue and red light have important effects on apple growth, carbon and nitrogen metabolism. These findings may be useful for determining the ideal light conditions for apple cultivation to maximize fruit yield and quality.

**Supplementary Information:**

The online version contains supplementary material available at 10.1186/s12870-021-03262-5.

## Background

Light, which is one of the most important environmental factors affecting plant growth and development, is an essential source of energy for plant activities; it also influences plant morphogenesis, photosynthesis, metabolism, and signal transduction [1]. Plants can sense changes in environmental light conditions, produce the corresponding light signals, and respond to light signals through their complex signal transduction pathways, thereby regulating growth and development [2]. Photoreceptors enable plants to sense changes in the external light environment [3]. To date, the photoreceptors that have been identified can be roughly divided into phytochrome, cryptochrome, phototropin, and ultraviolet-B (UV-B) photoreceptors [4, 5]. More specifically, phytochrome photoreceptors mainly sense red light and far-red light, cryptochrome and phototropin photoreceptors sense blue light, and UV-B photoreceptors sense UV-B light [5–7]. Many photoreceptor signal transduction models for different light signals have been established. Signaling cascades and transcriptional regulatory networks combine to complete the light response process and regulate plant development [6, 8].

Light quality significantly affects photosynthesis-related activities, including chloroplast formation, photosynthetic pigment synthesis, stomatal movement, leaf extension, and carbon assimilation [9]. The solar radiation spectrum is mostly between 300 and 2,600 nm, with wavelengths between 380 and 720 nm directly influencing plant photosynthetic activities. Additionally, the quality of photosynthetically active radiation can regulate plant photosynthesis, especially red light (600–700 nm) and blue light (400–500 nm) [10]. Previous studies revealed that red light can effectively promote leaf development, stem elongation, and root morphogenesis to increase plant growth [11]. It can also induce the accumulation of photosynthetic products by regulating photosynthetic organ development [12]. In contrast, blue light can significantly inhibit leaf growth, while also significantly promoting chlorophyll synthesis and stomatal conductance (Gs), thereby improving the net photosynthetic rate (Pn) of leaves [13, 14].

Many recent studies have examined the effects of light quality on tomato, cucumber, lettuce, ginger, and other vegetable crops [13, 15–17]. On the basis of the results of these studies, the light conditions used for cultivating vegetable crops under artificial light have been optimized, which has significantly increased the profitability of vegetable production. Compared with the number of investigations regarding vegetable crops, there have been fewer studies on the effects of light quality on perennial fruit trees, with minimal related research regarding secondary metabolism. Recent studies proved that blue light can promote anthocyanin synthesis in pear and grape fruits [18, 19] as well as chlorogenic acid synthesis in strawberry fruit [19]. Moreover, ultraviolet (UV) light is involved in the anthocyanin synthesis in grape, apple, pear, peach, and other fruit tree species [21–24]. However, the effects of light quality on the photosynthetic characteristics and carbon and nitrogen metabolism of fruit trees remain relatively unknown.

Apple (*Malus domestica* Borkh.), which is one of the four major fruit species worldwide, is characterized by a wide distribution, multiple varieties, a long history of cultivation, and fruit that are rich in nutrients [25]. Light has a significant effect on apple fruit coloration, with UV light promoting anthocyanin synthesis in the apple fruit peel [24, 26]. However, there are still few studies on the effects of different light qualities on the growth and development of apple seedlings. In this study, we explored the effects of red and blue light treatments on the growth and development of apple seedlings. Significant differences were detected in the photosynthetic characteristics, leaf chloroplast ultrastructure, chlorophyll fluorescence parameters, and carbon and nitrogen metabolism of apple leaves after red and blue light treatments. Furthermore, a high-throughput RNA sequencing (RNA-seq) analysis was conducted to compare the transcriptomes of apple seedlings cultured under blue, red, or white light. Because of the diversity in the development of apple production sites, clarifying the effects of different light qualities on apple photosynthesis, physiological characteristics, and fruit quality is critical for determining the ideal light conditions that will improve apple production in terms of fruit quality and yield.

## Results

### Effects of red and blue light treatments on apple plant growth

**Fig. 1:**
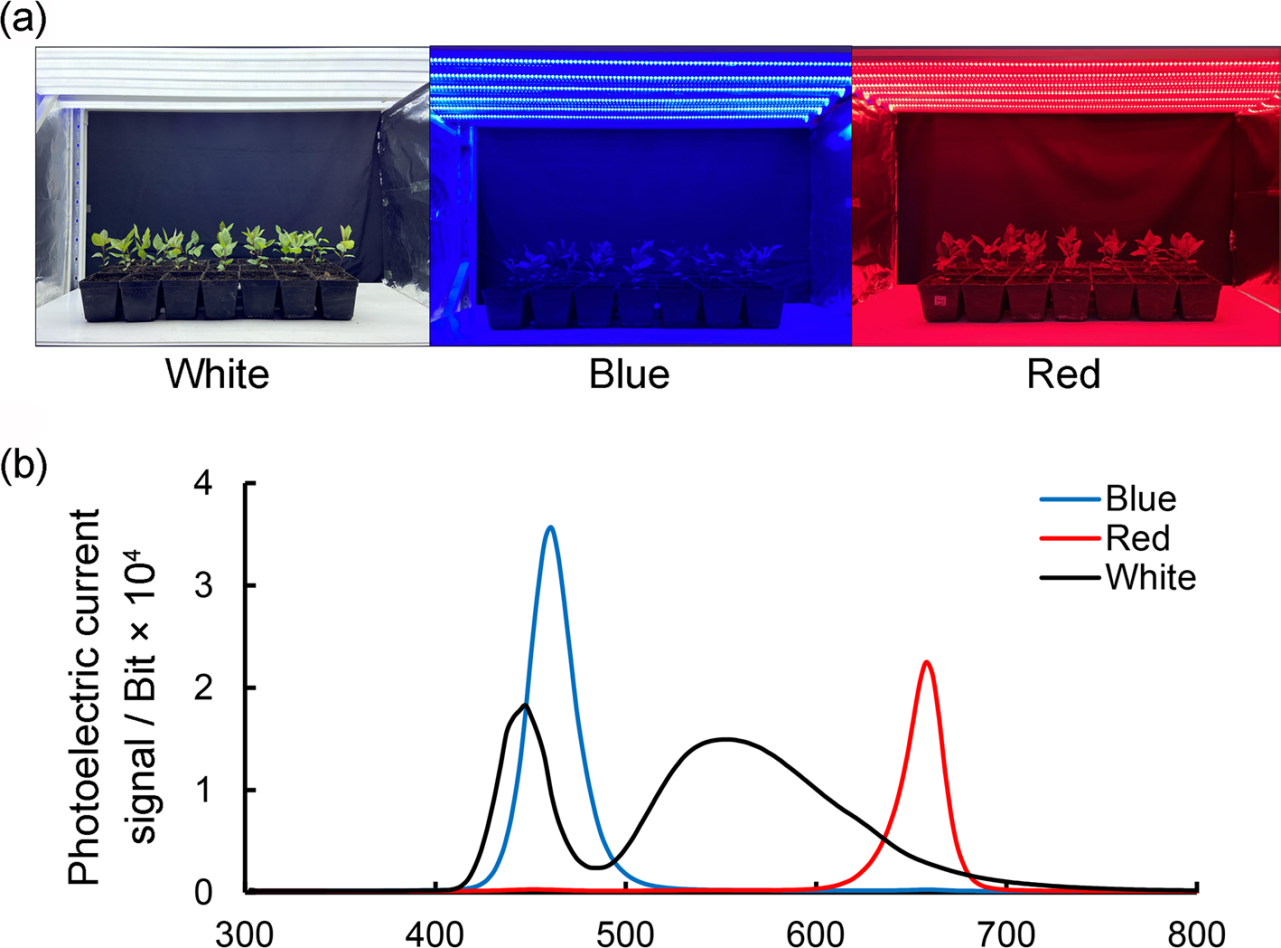
Treatment of apple seedlings under different light quality conditions. **a** Plants growing in the growth chamber under different light quality. **b** Light spectra of different treatments: blue light with a maximum peak emission at 446 nm, red light with a maximum peak emission at 664 nm

**Fig. 2:**
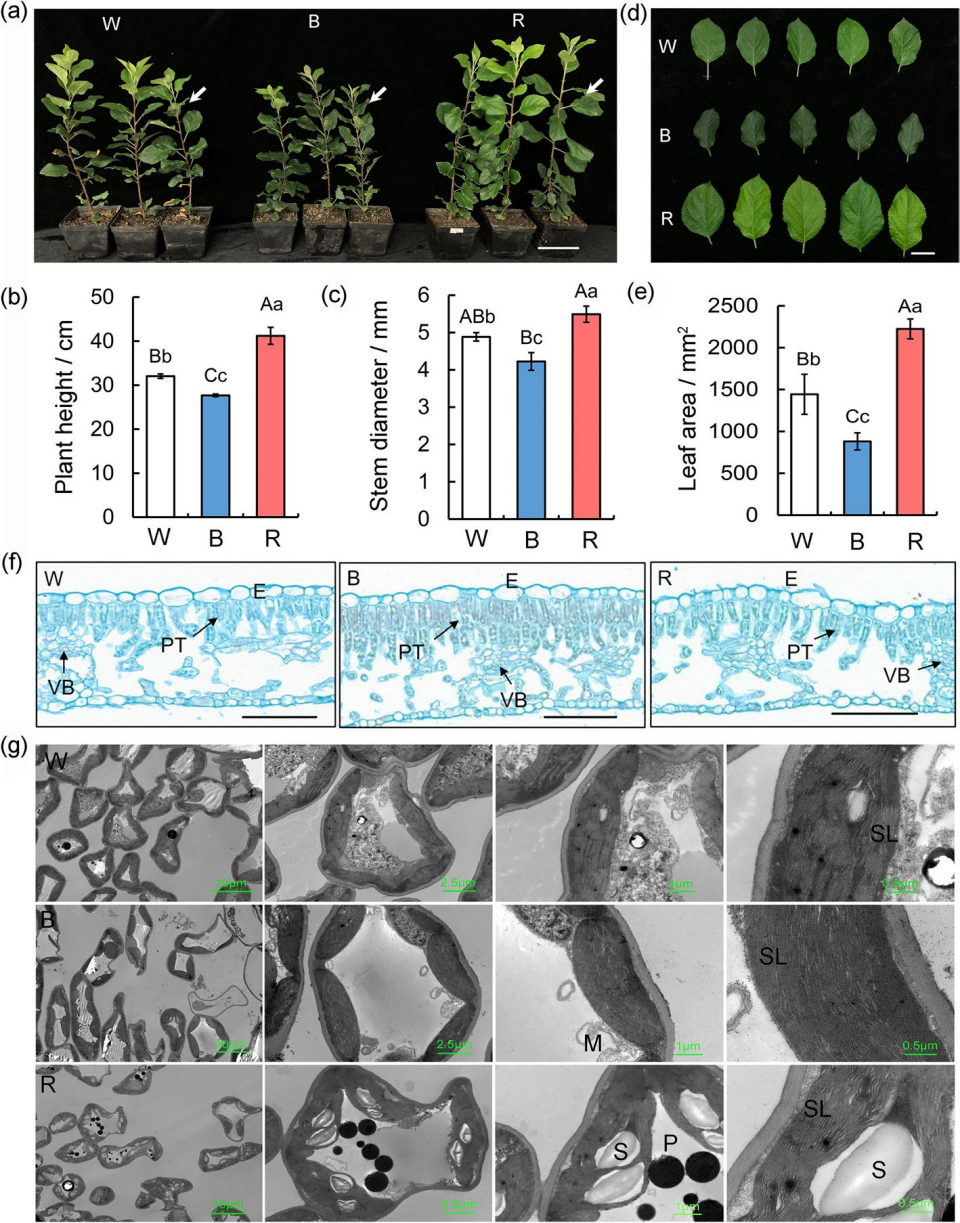
Effects of Blue and Red Light Treatments on the Growth and Leaves Development of Apple seedlings. **a** Growth status of apple seedlings after 30 days of different light quality treatments. Scale bars = 10 cm **b** Plant height, **c** stem thickness and **e** leaf area after of apple seedlings after 30 days of different light quality treatments. Values are means ± SD of three independent biological replicates. The uppercase letter indicates a very significant difference at the 1 % level (P < 0.01), and the lowercase letter indicates a significant difference at the 5 % level (P < 0.05). **d** Comparison of apple leaf morphology after 30 days of different light quality treatments. The leaves were taken from the third to fifth functional leaves, as shown by the arrows in Fig. 2a. Scale bars = 2 cm. **f** Leaf anatomy of apple leaves treated with different light quality. E: epidermis; PT: palisade tissue; VB: vascular bundle; W: white light; B: blue light; R: red light. Scale bars = 100 μm. **g** Chloroplast ultrastructure of apple leaves under different light conditions. The bars shown are 10 μm, 2.5 μm, 1 μm, and 0.5 μm from left to right respectively. M: Mitochondria; S: Starch grain; O: osmiophilic granules; SL: stroma lamella

Uniformly growing apple seedlings were incubated under white, blue, or red light (Fig. 1a). The wavelength peak of each light treatment was essentially as expected (Table S1), and the spectral parameters satisfied the experimental requirements (Fig. 1b). The apple plant growth status differed significantly among the light treatments (Fig. 2a). Compared with the white light treatment, the plant height, stem thickness, and leaf area of apple seedlings increased by 1.29, 1.12, 1.54 times after the red light treatment, and decreased by 13.6 %, 13.5 %, and 39.0 % after the blue light treatment. (Fig. 2b–e). The red light treatment clearly promoted plant growth, leaf growth, and stem elongation and thickening, whereas the exposure to blue light inhibited plant growth.

A spectral scan of the apple root system revealed that the total root length, root volume, and number of lateral roots of plants treated with red light were respectively 1.3-, 1.7-, and 1.6-times greater than the corresponding data for the plants treated with white light. There were no significant differences in the root indices between the plants treated with blue light and the plants treated with white light (Table 1). Thus, the red light treatment promoted the development of the apple root system, whereas the blue light treatment had no effect.

**Table 1:** Effects of light quality on apple roots

Light quality	Total root length (cm)	Root volume (cm^3^)	Number of root tips	Root surface area (cm^3^)	Average root diameter(mm)	Number of side roots
**White-light**	218.74±20.27b	1.37±0.16b	2397.67±223.12b	15.99±0.98a	0.54±0.05a	602.67±47.94b
**Blue-light**	227.79±8.28ab	1.64±0.27ab	3196.67±113.06a	15.82±0.37a	0.55±0.03a	816.67±123.51ab
**Red-light**	282.31±17.59a	2.29±0.27a	2804.67±221.99ab	16.89±1.17a	0.60±0.02a	993.67±34.64a

Significant differences are indicated by different lowercase letters (P < 0.05)

### Leaf anatomy and chloroplast ultrastructure of apple seedlings under different light conditions

Safranin-O and Fast-Green staining revealed tissue structural differences between the leaves treated with red light and the leaves treated with blue light. After the blue light treatment, the apple leaves had tightly arranged palisade tissues with dense chloroplasts, which is beneficial for light absorption and may lead to increased photosynthetic efficiency (Fig. 2f). This result is consistent with the result presented in Fig. 2d. As a consequence of the tight arrangement of the palisade tissue cells, the leaves treated with blue light were relatively small and dark green. In contrast, the leaves treated with white or red light had loosely organized palisade tissues.

The size and shape of the chloroplasts in the mesophyll cells were examined by transmission electron microscopy. In terms of morphology, chloroplasts developed best in response to the blue light treatment. More specifically, the chloroplasts under blue light were oblong. Additionally, the basal lamellae were stacked compactly, the stromal lamella structure was clear, and the chloroplast envelope was clear and complete, indicative of an increase in the light-capturing ability and in the energy conversion efficiency of the photosynthetic membrane. After the red light treatment, the chloroplasts tended to be short, thick, and swollen, with neatly and tightly stacked basal grana and many large starch grains. Additionally, compared with the effects of red light, the blue and white light treatments significantly decreased the number of osmophilic particles in chloroplasts (Fig. 2g).

### Effects of different light treatments on apple photosynthetic characteristics

**Fig. 3:**
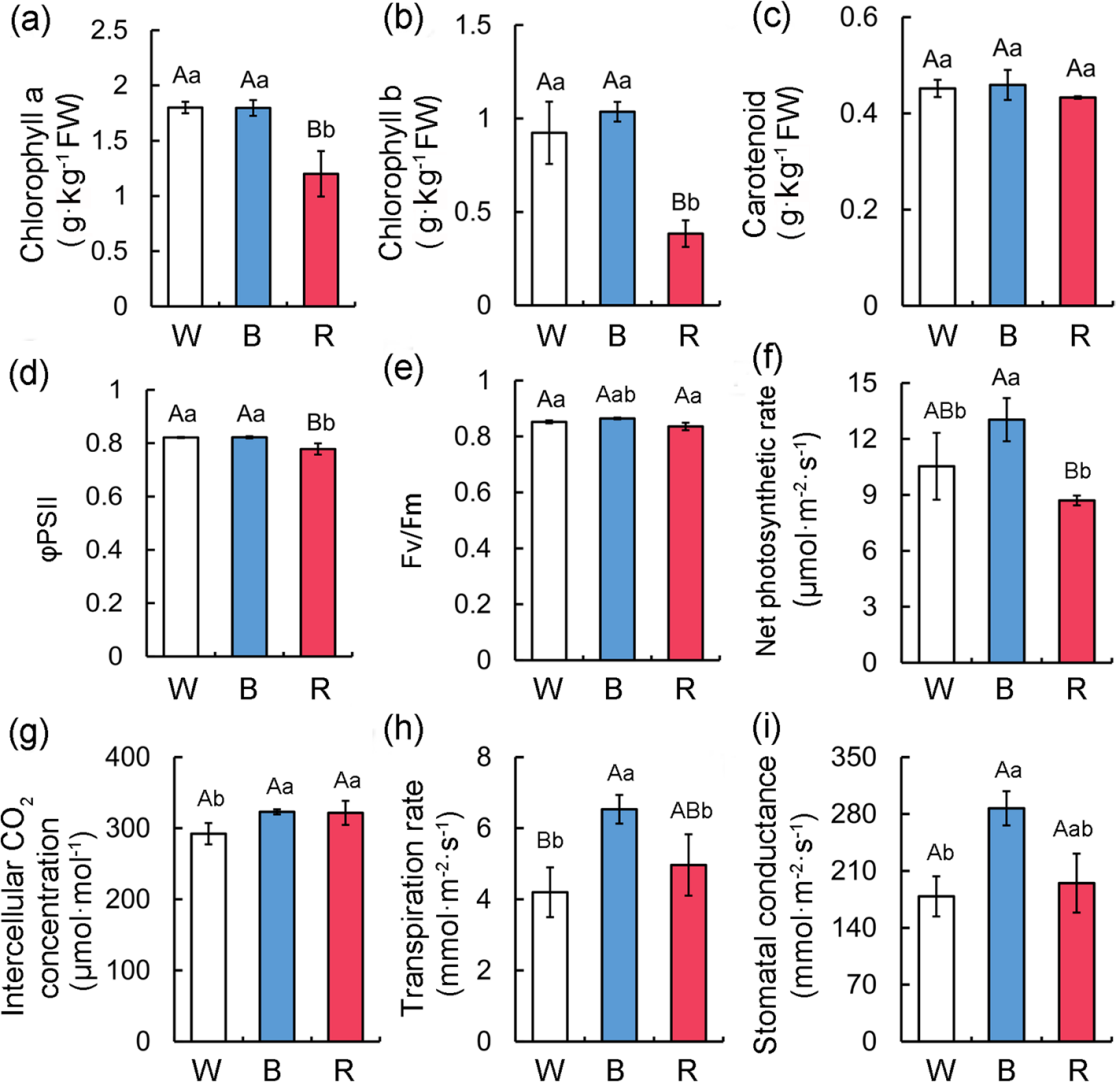
Effects of different light quality on photosynthetic characteristics of apple seedlings. **a** Chlorophyll a, **b** chlorophyll b and **c** carotenoid content in apple leaves after different light quality treatments. Chlorophyll fluorescence parameters of apple seedlings under different light conditions, including: **d** actual photochemical efficiency (ΦPSII); **e** the maximum photochemical efficiency of PSII under dark adaptation (Fv/Fm); **f** net photosynthetic rate; **g** Intercellular CO_2_ concentration; **h** Transpiration rate and **i** Stomatal conductance. Values are means ± SD of three independent biological replicates. The uppercase letter indicates a very significant difference at the 1 % level (P < 0.01), and the lowercase letter indicates a significant difference at the 5 % level (P < 0.05)

The light treatments in this study differentially affected the photosynthetic characteristics of apple seedlings. Regarding photosynthetic pigments, the chlorophyll *a* and *b* contents were significantly higher in the leaves treated with white or blue light than in the leaves treated with red light (Fig. 3a, b). Consistently, the total chlorophyll content in leaves treated with white light or blue light was also significantly higher than that of red light. However, there were no significant differences in the leaf carotenoid contents among the light treatments (Fig. 3c). Thus, red light is not conducive to apple leaf chlorophyll accumulation. In terms of chlorophyll fluorescence parameters, ΦPSII was significantly lower after the red light treatment than after the white and blue light treatments (Fig. 3d). In contrast, the different light treatments did not result in a significant difference in Fv/Fm (Fig. 3e). These results suggested that the red light treatment is not conducive to the photosynthesis of apple seedlings. Notably, the net photosynthetic rate (Pn), stomatal conductance (Gs), and transpiration rate (Tr) of apple leaves were significantly or extremely significantly higher following the blue light treatment than after the red and white light treatments (Fig. 3f–i), implying an exposure to blue light can increase leaf Gs and Tr, while improving photosynthetic efficiency.

### Effects of different light treatments on carbon and nitrogen metabolism

**Fig. 4:**
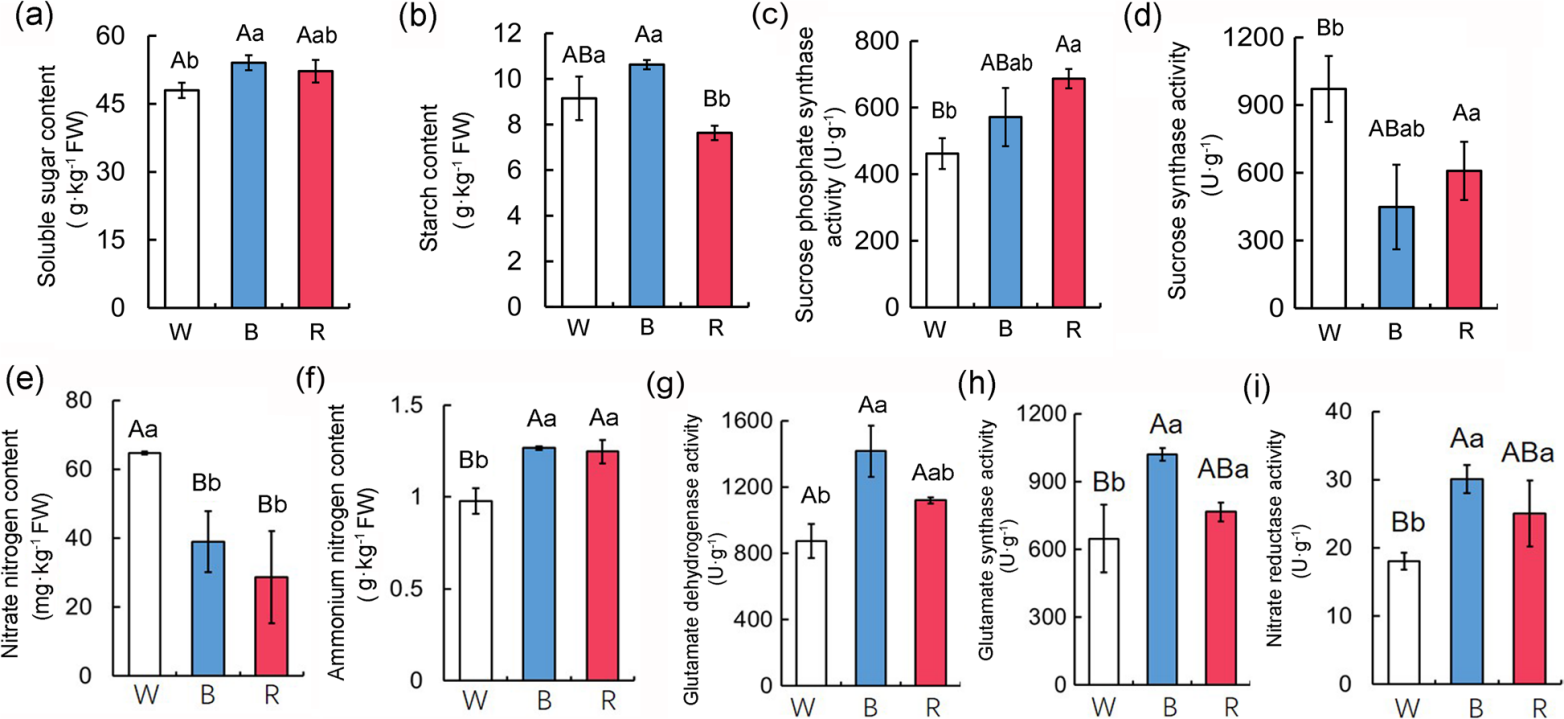
Effects of Different Light Quality on Carbon and Nitrogenous metabolism. **a** and **b** The content of soluble sugar and starch in apple leaves after different light quality treatments. **c** and **d** Sucrose phosphate synthase (SPS) and Sucrose synthase (SS) activities in apple seedlings under different light quality. **e** and **f** The content of nitrate nitrogen and ammonium nitrogen in apple leaves after different light quality. **g** to **i** Glutamate dehydrogenase (GDH), glutamate synthase (GOGAT), and nitrate reductase (NR) activities in apple seedlings under different light quality. Values are means ± standard deviation (SD) of three independent biological replicates. The uppercase letter indicates a very significant difference at the 1 % level (P < 0.01), and the lowercase letter indicates a significant difference at the 5 % level (P < 0.05)

Light quality influenced the accumulation of photosynthetic products and the activities of key enzymes mediating carbon metabolism in apple seedlings. The soluble sugar content was significantly higher in the leaves treated with blue light than in the leaves treated with white light (Fig. 4a). The starch content was significantly lower in the leaves treated with red light than in the leaves treated with blue or white light (Fig. 4b). Additionally, sucrose phosphate synthase (SPS) activity was highest in the leaves treated with red light, followed by the leaves treated with blue light (Fig. 4c). In contrast, sucrose synthase (SS) was more active under white light than under either red or blue light (Fig. 4d). These results suggest that an exposure to blue light positively affects the accumulation of soluble sugars and photosynthetic products, whereas an exposure to red light promotes SPS activity.

The light treatments differentially affected the nitrate nitrogen and ammonium nitrogen contents in apple leaves as well as the activities of key enzymes contributing to nitrogen metabolism. The nitrate nitrogen content was lower in the leaves treated with red or blue light than in the leaves treated with white light (Fig. 4e). The opposite pattern was observed for the ammonium nitrogen content in apple leaves (Fig. 4f). Moreover, the activities of key enzymes involved in nitrogen metabolism, i.e., dehydrogenase (GDH), glutamate synthase (GOGAT), and nitrate reductase (NR), were significantly higher after the blue light treatment than after the white light treatment (Fig. 4 g–i). Therefore, the blue and red light treatments enhanced the accumulation of ammonium nitrogen in apple seedlings. Furthermore, the blue light treatment significantly promoted nitrogen metabolism.

### Analysis of genes differentially expressed following the blue, red, and white light treatments by RNA-seq

**Fig. 5:**
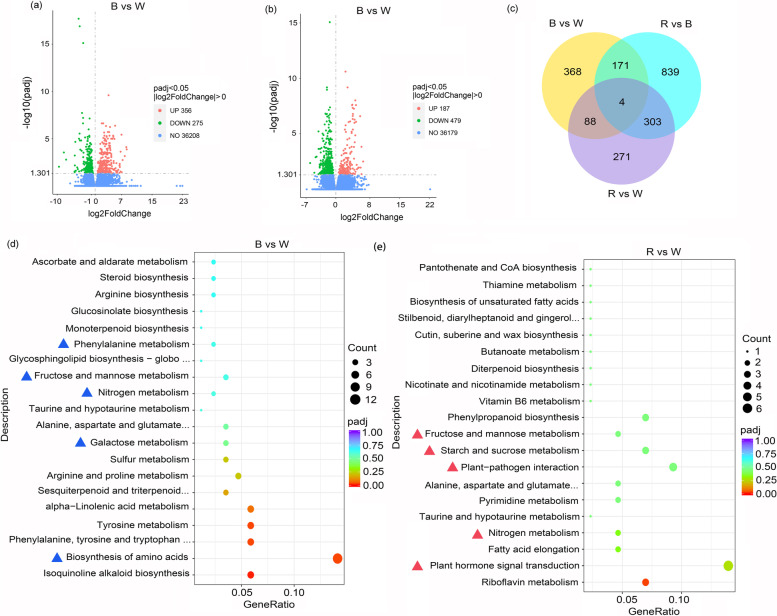
RNA-seq Analysis and and screening of differentially expressed genes (DEGs) between Blue-/red-light and white-light treatment. **a** DEGs between blue light treatment and white light control. **b** DEGs between red light treatment and white light control. **c** The Venn diagram of the DEGs between the blue, red, and white light treatments. **d** KEGG pathway enrichment analyses of up-regulated DEGs between blue light and white light treatment. **e** KEGG pathway enrichment analyses of up-regulated DEGs between red light and white light treatment

To analyze the differentially expressed genes (DEGs) related to the blue and red light treatments, the RNA-seq transcriptome profiles of the apple leaves treated with blue, red, or white light were analyzed. A comparison between the leaves treated with blue light and the leaves treated with white light revealed 631 DEGs, of which 356 and 275 genes were respectively expressed at higher and lower levels in the leaves treated with blue light (Fig. 5a; Table S2). A comparison between the leaves treated with red light and the leaves treated with white light resulted in the detection of 666 DEGs, of which 187 and 479 genes were respectively expressed at higher and lower levels in the leaves treated with red light (Fig. 5b; Table S2). A Venn diagram of the DEGs indicated 88 DEGs were common to the blue and red light treatments (Fig. 5c).

The DEGs were functionally characterized on the basis of the enriched Kyoto Encyclopedia of Genes and Genomes (KEGG) pathways. The DEGs with up-regulated expression in the leaves exposed to blue light (relative to their expression levels in the white light-treated leaves) were primarily associated with the biosynthesis of amino acids, galactose metabolism, nitrogen metabolism, and fructose and mannose metabolism, implying blue light promotes carbon and nitrogen metabolism in apple leaves (Fig. 5d). Some DEGs were related to phenylalanine metabolism, suggesting that blue light can also promote secondary metabolism. The DEGs with up-regulated expression in the leaves exposed to red light (relative to their expression levels in the white light-treated leaves) were mainly involved in plant hormone signal transduction, plant–pathogen interactions, starch and sucrose metabolism, fructose and mannose metabolism, and nitrogen metabolism, indicating red light induces carbon and nitrogen metabolism and influences hormone signaling and plant disease resistance (Fig. 5e). The raw RNA-seq data were deposited in the NCBI sequence read archive (accession number PRJNA735024).

## Screening of up-regulated DEGs and qRT-PCR validation

**Fig. 6:**
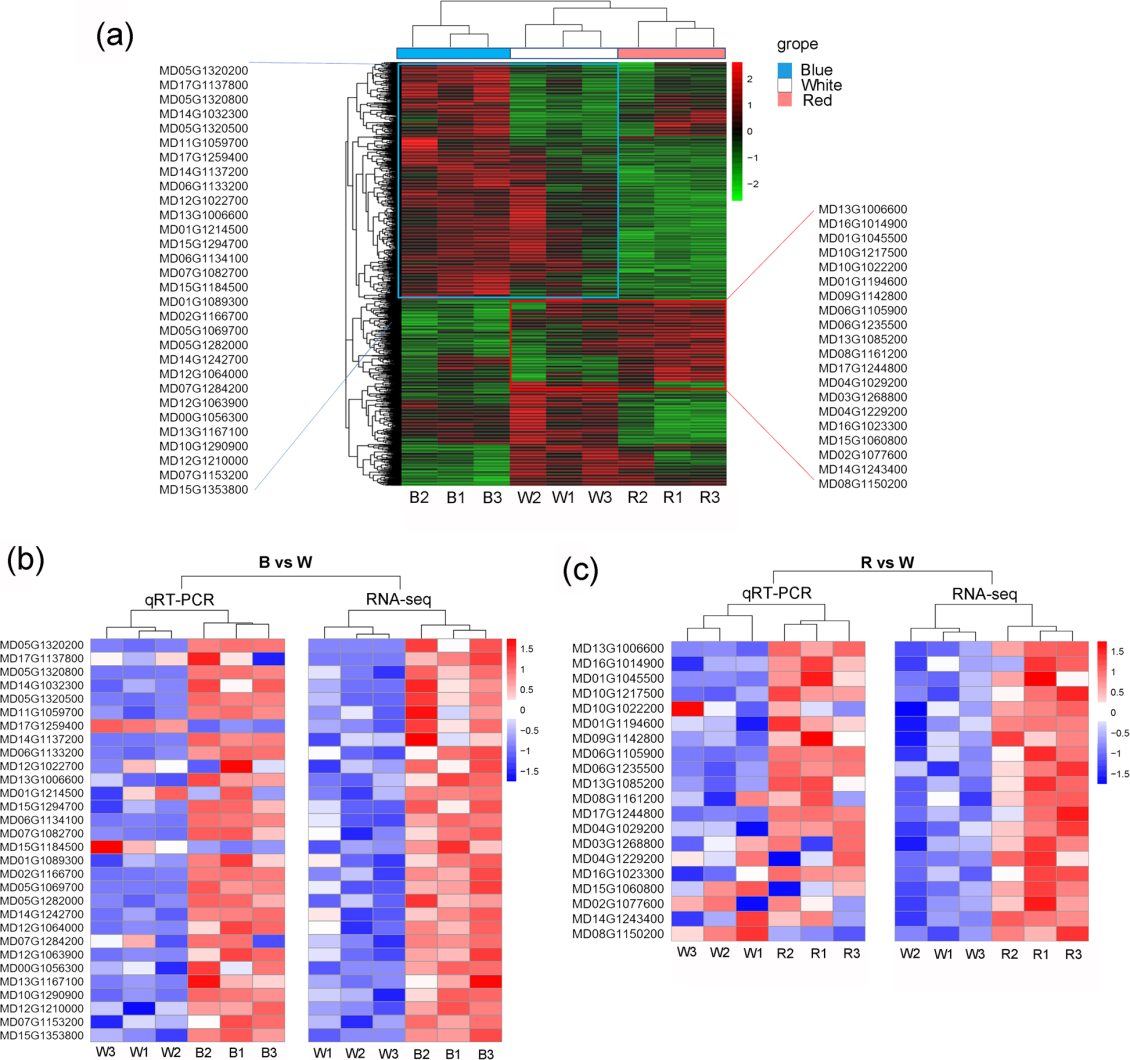
The screening of up-regulated DEGs and qRT-PCR validation. **a** Cluster analysis on the DEGs of all comparison groups. Blue box: Clusters of DEGs up-regulated after blue light treatment; Red box: Clusters of DEGs up-regulated after red light treatment. A total of 30 up-regulated genes were screened in blue-light treatment apple seedlings, and 20 up-regulated genes in red-light treatment. **b** and **c** Heatmap analysis compared the qRT-PCR verification and RNA-seq results of 30 DEGs selected in blue-light treatment and 20 DEGs selected in red-light treatment. *MdActin* was used as internal control gene

To further investigate the differences in the expression patterns resulting from the various light treatments, the DEGs in all comparison groups were included in a cluster analysis (Fig. 6a). Up-regulated gene expression modules were detected for the blue and red light treatments (Fig. 6a). A total of 30 and 20 up-regulated genes were detected in the blue light- and red light-treated apple seedlings, respectively (relative to their expression levels in the white light-treated leaves). These genes encode diverse enzymes and photosynthesis-related proteins, including glutamine synthetase (MD13G1006600), glutamate decarboxylase (MD14G1242700 and MD06G1235500), chlorophyll *a*/*b* binding protein (MD17G1137800), cytochrome P450 (MD11G1059700, MD17G1259400, and MD12G1022700), and UDP-glycosyltransferase (MD04G1029200). They also encode transcription factors (TFs), including those belonging to the bHLH (MD14G1137200, MD06G1133200, MD01G1089300, MD12G1064000, MD12G1063900, and MD01G1045500), MYB (MD07G1153200), AP2/ERF (MD14G1032300 and MD10G1290900), and NAC (MD10G1217500) families (Table S3).

A qRT-PCR analysis was performed to validate the DEGs detected by RNA-seq. The qRT-PCR data were mostly consistent with the RNA-seq results (Fig. 6b, c). However, MD12G1022700, MD01G1214500, MD07G1284200, MD03G1268800, and MD04G1229200 were identified as DEGs on the basis of the RNA-seq results, but the qRT-PCR data suggested they were not differentially expressed. Moreover, the opposite expression patterns were detected by the qRT-PCR and RNA-seq analyses for MD17G1259400, MD15G1184500, and MD08G1150200. Notably, the qRT-PCR and RNA-seq analyses both indicated MD14G1137200, which encodes a MYC2-like protein, is expressed at much higher levels in apple leaves treated with blue light than in apple leaves treated with white light (i.e., 30.72-fold and 22.32-fold higher according to the qRT-PCR and RNA-seq data, respectively).

## Discussion

The solar radiation spectrum plants are exposed to differs among regions and between seasons [27]. Elucidating the effects of different light qualities on crop growth and development may be relevant for selecting cultivation sites with light conditions that will optimize crop yield and quality. In this study, we observed that blue light significantly inhibits apple plant growth and leaf extension, but it promotes the development of leaf tissue structures and chloroplasts. Although an exposure to red light can enhance apple plant growth and root development, it will also result in loosely organized leaf palisade tissues and low chlorophyll contents. These findings are consistent with the results of earlier studies on other plant species. For example, in a recent investigation, the hypocotyl length, plant height, internode distance, and leaf area of tomato seedlings were adversely affected by blue light [28]. Another recent study revealed that compared with lettuce plants grown under monochromatic red light, plants exposed to an increasing proportion of blue light exhibit inhibited growth (e.g., stem growth), but their chloroplast abundance and single-leaf photosynthetic efficiency may increase [29]. In rapeseed, increasing the ratio of blue light can effectively increase leaf thickness, leaf mass per unit area, and chlorophyll content per unit weight, while also leading to the development of tightly organized leaf palisade tissues [30], which is consistent with the observations in the current study. In contrast, in lettuce and Arabidopsis, stem growth resulting from red light irradiation is significantly greater than that induced by other treatments [31, 32], which is also consistent with the results of this study. However, there are also reports that blue light can promote plant growth and leaf extension better than red light [33–35], indicating that the effects of blue and red light treatments on plant growth vary among species.

Light quality changes can alter plant photosynthetic rates by affecting chloroplast development. In cucumber, blue light promotes chloroplast development, leading to extensive layer stacking and minimal starch accumulation [36]. The chloroplast ultrastructural integrity in upland cotton seedlings is positively affected by blue light irradiation, which results in a clearly visible layered structure [37]. Consistent with this earlier finding, we observed that the chloroplasts of apple leaves treated with blue light developed well in terms of their ultrastructural features, which were revealed by electron microscopy. These chloroplasts were oblong, and their stroma lamellae were clear and densely arranged. Additionally, previous research proved that red light can significantly promote the accumulation of starch granules in chloroplasts [38] and that the accumulation of starch granules is likely detrimental to photosynthetic activities in plant leaves [39]. In the current study, we determined that a red light treatment can lead to the accumulation of starch granules in chloroplasts.

In addition to the chloroplast structure, photosynthetic pigment contents (e.g., chlorophyll) directly affect the photosynthetic efficiency of plants to a certain extent. In strawberry, the chlorophyll content and Fv/Fm are positively correlated with the red light: blue light ratio, but they are negatively correlated with the chlorophyll *a*/*b* contents [40]. In lettuce, the chlorophyll content is higher in plants exposed to blue light than in plants exposed to red light [41]. Similar results were reported for spinach and Orchidaceae species [42, 43]. The data generated in the present study indicate that blue light promotes the synthesis of chlorophyll *a* and *b* and increases Gs and Pn, whereas red light decreases the chlorophyll content and ΦPSII.

Carbon and nitrogen metabolism is the most basic physiological metabolic process associated with plant growth and development [44]. Many studies have confirmed that light quality can affect plant carbon and nitrogen metabolism. In cabbage, a red light treatment is conducive to the accumulation of carbon metabolites such as soluble sugars, sucrose, and starch [45]. However, in lettuce, the leaf soluble sugar, starch, carbohydrate, and sucrose contents are significantly higher under red–blue light than under monochromatic red light [46]. In tomato, red, blue, and red–blue light treatments can significantly increase SS activity, whereas red and blue light treatments have the opposite effect on SPS activity [47]. The results of the current study suggest that a blue light treatment promotes the accumulation of soluble sugars in photosynthetic products, whereas an exposure to red light leads to increased SPS activity. Moreover, a previous study confirmed that a blue light treatment decreases NR activity and the nitrate nitrogen content, but significantly increases GS and GOGAT activities, whereas a red light treatment significantly decreases NR, GS, and GOGAT activities [47]. We observed that both blue and red light treatments decrease the nitrate nitrogen content, increase the ammonium nitrogen content, and promote NR and GOGAT activities.

The effects of light quality on plant growth and development have recently been investigated via transcriptomic and other omic-based approaches. In strawberry, metabolomic and transcriptomic analyses revealed that blue light can promote *FvHCT* expression and chlorogenic acid synthesis [20]. In tea plants, a transcriptomic analysis demonstrated that high-intensity blue light induces several pathways related to photosynthesis, lipid metabolism, and flavonoid synthesis. Moreover, the TFs encoded by the DEGs detected during an earlier comparison of the effects of different light treatments were mostly from the bHLH and MYB families [48]. In accordance with the results of earlier studies, our transcriptomic analysis indicated that blue light can promote amino acid biosynthesis and carbon and nitrogen metabolism in apple. Furthermore, we identified some DEGs encoding bHLH, MYB, AP2/ERF, NAC, and other TFs. We have performed qRT-PCR verification on the selected DEGs, and the results are mostly consistent with the RNA-seq, except for a few genes. We speculate that this inconsistency is caused by large sequencing errors caused by the low expression abundance of the gene itself, or it may be caused by the interference of homologous gene expression. Specific bHLH TFs, designated PHYTOCHROME-INTERACTING FACTOR (PIF) TFs, including PIF1 and PIF3, reportedly negatively regulate chlorophyll biosynthesis and light responses [49, 50]. Another bHLH TF, MYC2, contributes to blue light-mediated Arabidopsis seedling development [51]. In this study, we identified a gene encoding a MYC2-like protein. The expression of this gene was significantly up-regulated in response to blue light. Its potential role related to plant development will need to be determined in future studies.

## Conclusions

**Fig. 7:**
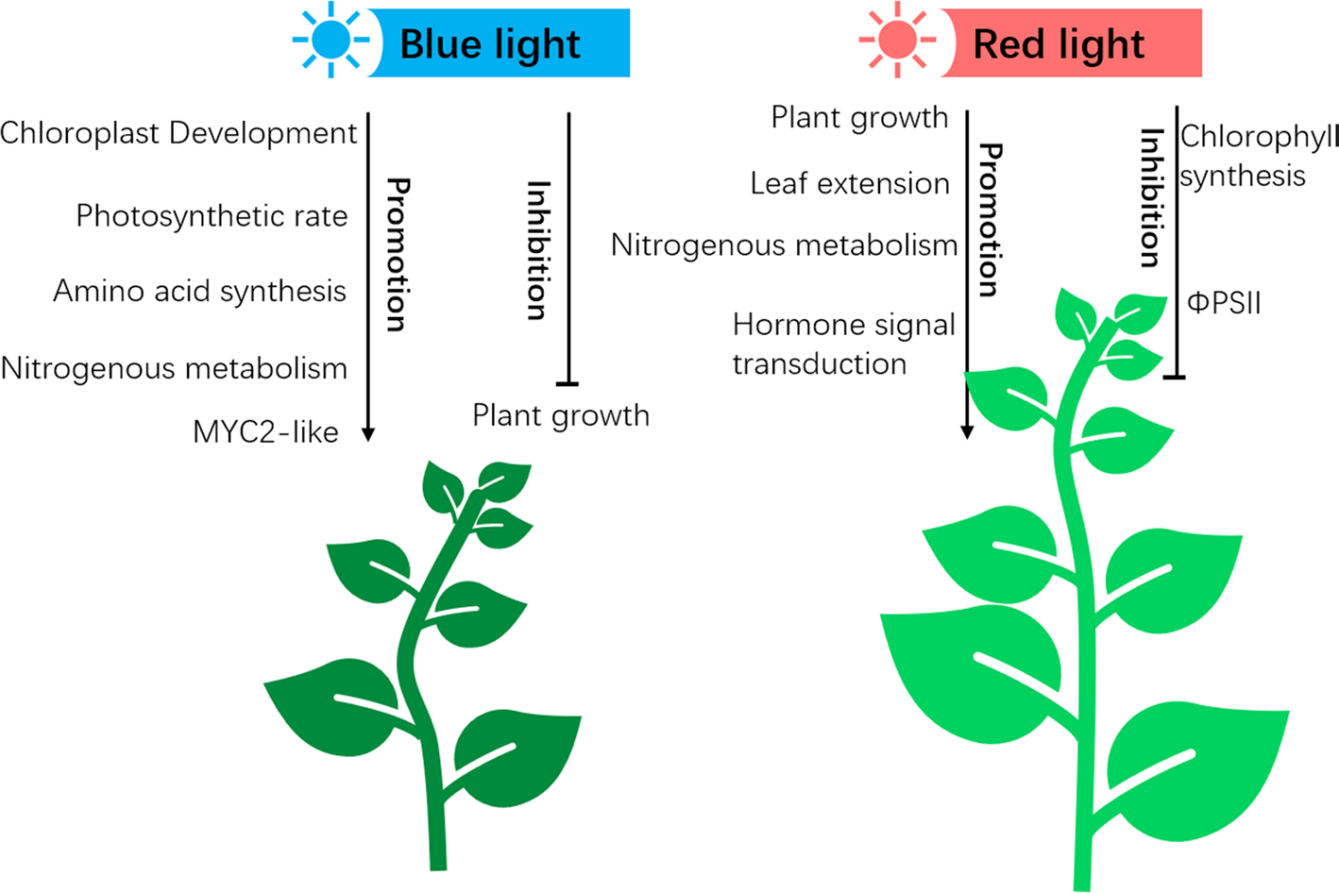
Proposed model of how blue light and red light affect the growth and development of apple seedlings

In conclusion, blue light has a significant inhibitory effect on apple plant growth and leaf extension, but it positively affects the leaf tissue structure and chloroplast development, while also increasing Gs, Tr, and photosynthetic efficiency. A red light treatment can promote apple plant growth and root development, but it can also lead to loosely organized leaf palisade tissues and low chlorophyll contents. Blue and red light treatments can enhance the accumulation of ammonium nitrogen in apple seedlings. In particular, an exposure to blue light significantly induces nitrogen metabolism (Fig. 7). The RNA-seq analysis in this study indicated both blue light and red light can significantly up-regulate the expression of genes related to carbon and nitrogen metabolism. Furthermore, blue light can increase amino acid biosynthesis and flavonoid metabolism, whereas red light can promote plant hormone signal transduction.

## Methods

### Plant materials and light treatments

Due to the self-incompatibility of apples, the seedlings obtained from seed germination have a wide range of character separation. Therefore, this study used apple tissue culture seedlings with absolutely consistent genetic background as the test material. An offspring of apple (*Malus domestica*) ‘Royal Gala’ that gives higher regeneration capacity, named ‘GL-3’ was used for light treatments. ‘GL-3’ apple seedlings have been subcultured and cultivated by our laboratory [52]. Potted ‘GL-3’ plants that were growing robustly and uniformly were placed in a growth room for the light treatments. The plants were initially incubated under the following conditions: 16-h light (24 °C)/8-h dark (18 °C) cycle; light intensity of 186 ± 9.8 µmol m^−2^ s^−1^ at 40 cm under the light source; and relative humidity of 60 ± 2.8 %. The plants were watered once every 3 days. To expose the plants to different light qualities, they were irradiated with white (i.e., control), red, or blue light, with 20 apple seedlings per treatment. The UNISPEC-DC™ spectrum analyzer (PP-SYSTEMS, Amesbury, US) was used to record the spectrum data for each light treatment. Specifically, the waveband was 300–800 nm and the scanning wavelength interval was 3.3 nm.

#### Examination of morphological and physiological characteristics

After a 30-day exposure to different light qualities, the apple seedlings are completely lignified and have enough functional leaves (10-12 layers). The plant height, stem thickness, leaf area, and root configuration were analyzed. For each treatment, 10 apple seedlings were collected as biological replicates. Plant height was measured by recording the height from the base of the first petiole to the highest stem bifurcation. Stem thickness was determined by measuring the diameter of the stem base. The functional leaves (third to fifth leaves from the top) were are used to determine the leaf area. Leaf area was calculated using the Yaxin-1241 leaf area meter (YAXIN-LIYI, Beijing, China). A root spectral scanning imaging analysis system (SpectraScan-R, Beijing, China) was used to determine the total root length, root surface area, average root diameter, and number of lateral roots.

### Analysis of the leaf anatomy and chloroplast ultrastructure

The functional leaves (third to fifth leaves from the top) collected from plants that underwent different light treatments were dehydrated and embedded to make paraffin sections. The prepared sections were placed in xylene for 20 min, absolute ethanol for 20 min, and 75 % alcohol for 5 min, after which they were rinsed with water (i.e., dewaxing). The sections were then placed in Safranin-O staining solution for 2 h [53]. After washing off the excess dye, the sections were treated with 50 %, 70 %, and 80 % alcohol solutions for 5 s each. The decolorized sections were stained with Fast-Green staining solution for 15 s. Leaf anatomy was examined using the Eclipse E100 epifluorescence microscope (Nikon, Tokyo, Japan).

The functional leaves (i.e., the third to fifth leaves from the top) were cut into small pieces (1 mm × 1 mm) and immediately placed in a 2.5 % glutaraldehyde fixing solution. Air was evacuated using a vacuum pump until the leaf pieces sank to the bottom of the container. The samples were incubated at room temperature (25 °C) for 2 h and then stored at 4 °C. The samples were rinsed three times with 0.1 M phosphate buffer (pH 7.4) for 15 min each, fixed with 1 % osmic acid at room temperature for 7 h, and then rinsed three times with 0.1 M phosphate buffer as before. After dehydrating and embedding the leaf tissue samples, ultra-thin Sects. (60–80 nm) were prepared using the UC7 microtome (Leica, Wetzlar, Germany). The sections were stained and then examined using the HT7700 transmission electron microscope (Hitachi, Tokyo, Japan).

### Analyses of photosynthetic characteristics and chlorophyll fluorescence

After a 30-day exposure to different light qualities, the photosynthetic pigment contents of the functional leaves (third to fifth leaves from the top) were determined according to an ethanol/acetone extraction method. Briefly, fresh leaves (0.2 g) were placed in a 20 ml test tube containing an ethanol/acetone solution (v:v = 1:1) and incubated in darkness for 24 h. The absorbance of the solution was measured at 470 nm to determine the carotenoid content and at 645 and 663 nm to determine the chlorophyll content. The leaf photosynthetic pigment contents were calculated as previously described [17].

The Pn, intercellular CO_2_ concentration (Ci), Gs, and transpiration rate (Tr) of the functional leaves were measured using the CIRAS-3 photosynthetic apparatus (PP-SYSTEMS, Amesbury, US). The actual photochemical efficiency (ΦPSII) and the maximum photochemical efficiency (Fv/Fm) of the functional leaves were determined using a portable pulse-modulated fluorometer (Hansatech, Norfolk, UK).

### Determination of the sugar content and enzyme activities related to carbon metabolism

The soluble sugar content was determined according to a modified anthrone colorimetric method [54]. Specifically, 0.3 g leaf samples were added to 8 ml ddH_2_O and boiled for 30 min. This step was repeated twice. The resulting solutions were filtered and collected in a 25 ml volumetric flask to produce a constant volume. A 0.1 ml aliquot of the solution was mixed with 1.9 ml ddH_2_O in a 20 ml graduated test tube before 0.5 ml anthrone ethyl acetate reagent and 5 ml sulfuric acid were added (in this order). The resulting solution was mixed and immediately placed in boiling water for 2 min and then cooled to room temperature. The absorbance (at 630 nm) of the solution was measured using the UV-2450 spectrophotometer (Shimadzu, Tokyo, Japan). The remaining residue was added to 20 ml ddH_2_O, boiled for 15 min, and mixed with 2 ml 9.2 M perchloric acid. The solution was incubated for 15 min before using the same method to determine the starch content. Glucose (analytical reagent; Sigma Chemicals, Wuhan, China) was used as the master standard.

The activities of the carbon metabolism-related enzymes, namely sucrose synthase (SS) and sucrose phosphate synthase (SPS), were measured using commercially available assay kits (Solarbio, Beijing, China; catalog nos BC0580 and BC0600).

### Determination of the nitrogen content and enzyme activities related to nitrogen metabolism

The ammonium nitrogen and nitrate nitrogen contents of leaves were measured using commercially available assay kits (Solarbio, Beijing, China; catalog nos BC1520 and BC1500). The activities of nitrogen metabolism-related enzymes, namely glutamate dehydrogenase (GDH), glutamate synthase (GOGAT), and nitrate reductase (NR), were also measured using commercially available assay kits (Solarbio, Beijing, China; catalog nos BC1460, BC0070, and BC0080). The determination methods of GDH and GOGAT enzyme activity are similar. About 0.1 g leaf tissue was added to 1 mL extraction solution for ice-bath grinding, then centrifuged at 8000 g at 4 ℃ for 10 min, and the supernatant was taken and placed on ice for testing. Record the initial absorbance at 20 s and the absorbance at 5 min 20 s at a wavelength of 340nm. Enzyme activity is calculated based on the weight of the sample. The consumption of 1nmol NADH per g of tissue per minute is defined as an enzyme activity unit. The determination of NR enzyme activity requires induction treatment in advance. After extraction, the initial absorbance at 340nm wavelength and the absorbance after 30 min of reaction are measured.

### RNA-seq analysis

The functional leaves (third to fifth leaves from the top) collected from plants that underwent different light treatments were used for RNA-seq. Total RNA was extracted using the RNAprep Pure Plant kit (Tiangen, Beijing, China). After amplification and denaturation steps, a cDNA library was constructed and sequenced using the Illumina system (Illumina, SanDiego, US). Raw reads were filtered by removing low-quality reads containing adapters and unknown bases. The remaining clean reads were aligned to the apple reference genome sequence using the HISAT program [55]. The fragments per kilobase of transcript per million mapped reads (FPKM) value of each gene was calculated on the basis of gene length and the number of reads mapped to the gene. Differential gene expression was analyzed using the DESeq2 R package (1.20.0) [56]. Gene Ontology (GO) and KEGG enrichment analyses of the DEGs were performed using the clusterProfiler R package. The false discovery rate-adjusted P value was calculated. Additionally, FDR ≤ 0.05 was generally considered to indicate significant enrichment.

### Quantitative real-time (qRT)-PCR analysis

The functional leaves (third to fifth leaves from the top) collected from plants that underwent different light treatments were used for qRT-PCR analysis. First-strand cDNA was synthesized from 1 µg total RNA using the First-Strand cDNA Synthesis kit (Tiangen, Beijing, China). Primers were designed using the Beacon Designer 7 program and synthesized by Sangon Biotech (Shanghai, China). The qRT-PCR analysis was conducted using the SYBR Green PCR Master Mix (TransGen Biotech, Beijing, China) and the iCycler iQ5 system (Bio-Rad, Hercules, CA). The *MdActin* gene served as an internal control. Relative gene expression levels were calculated according to the 2^−ΔΔCt^ method of the IQ5 2.0 program [52]. The primers are shown in Table S4.

### Data analysis

All experiments were performed in triplicate. Error bars in the figures provided herein indicate the standard deviation of three replicates. Data are presented as the mean ± SD of three independent biological replicates. Significant differences were determined according to the *t*-test using GRAPHPAD PRISM 6.02 or the DPS software. Differences were considered significant when P < 0.05 or < 0.01.

## Supplementary Information

**Additional file 1: Table S1.** The photoeletric current signal of each light treatment.

**Additional file 2: Table S2.** Differentially expressed genes between blue/red and white light-treated apple seedlings.

**Additional file 3: Table S3.** Up-regulated genes detected in the blue light- and red light-treated apple seedlings.

**Additional file 4: Table S4.** The primers used for RT-qPCR.

## Data Availability

The raw sequencing data were deposited in NCBI Sequence Read Archive under the accession number PRJNA735024 (https://dataview.ncbi.nlm.nih.gov/object/PRJNA735024).
